# The origin and early evolution of cytokinin signaling

**DOI:** 10.3389/fpls.2023.1142748

**Published:** 2023-06-28

**Authors:** Anahid E. Powell, Alexander Heyl

**Affiliations:** Department of Research and Development, Garden City, NY, United States

**Keywords:** conquest of land, cytokinin, metabolism, signal transduction, phytohormone, evolution

## Abstract

Angiosperms, especially *Arabidopsis* and rice, have long been at the center of plant research. However, technological advances in sequencing have led to a dramatic increase in genome and transcriptome data availability across land plants and, more recently, among green algae. These data allowed for an in-depth study of the evolution of different protein families – including those involved in the metabolism and signaling of phytohormones. While most early studies on phytohormone evolution were phylogenetic, those studies have started to be complemented by genetic and biochemical studies in recent years. Examples of such functional analyses focused on ethylene, jasmonic acid, abscisic acid, and auxin. These data have been summarized recently. In this review, we will focus on the progress in our understanding of cytokinin biology. We will use these data to synthesize key points about the evolution of cytokinin metabolism and signaling, which might apply to the evolution of other phytohormones as well.

## Introduction

The conquest of land by plants is one of the major events in the evolution of life on earth. While the sparse fossil record makes it difficult to date landfall, recent analyses place this event around 550 mya ago (Morris et al., 2018; Fürst-Jansen et al., 2020). Not only did this step lead to a great diversification of morphology and physiology of the flora on this planet, but it also profoundly changed the environmental conditions of the terrestrial surface for all organisms (Kenrick and Crane, 1997; Waters, 2003). The major obstacles for the first land plants to overcome were probably primarily of a physical nature, such as drought, increased UV radiation, and the effect of gravity. But the first land plants were not alone, as bacteria and fungi had colonized the land (de Vries and Rensing, 2020). In fact, the “mycorrhizal landing” hypothesis states that the ability of early plants to form symbiotic relationships with mycorrhizal fungi was crucial to allow the first land plant to survive in this new habitat (reviewed in Delaux et al., 2015; Rensing, 2018). A wide variety of different photosynthetic organisms established themselves in terrestrial habitats (Hoffmann, 1989). However, only the *Streptophyte* algae and more specifically the last common ancestor of Zygnematophyceae and Embryophytes most likely gave rise to land plants (reviewed by de Vries and Archibald, 2018). This might seem surprising given the low complexity of the Zygnematophyceae body plan (Zhou and von Schwartzenberg, 2020). However, this simple morphology might have been very advantageous in mastering the new challenges (e.g., higher relative gravity due to lack of buoyancy, and lack of water) posed by the new terrestrial environment. On the molecular level, recently sequenced genomes of Zygnematophyceae confirmed earlier studies that pointed to a substantial gene gain in the later diverging clades of the charophytes. These gene gains have also been in phytohormone metabolism and signaling (de Vries et al., 2018; Cheng et al., 2019; de Vries and Rensing, 2020; Feng et al., 2023).

A functional system for at least some of the phytohormones has been considered crucial for the conquest of land (Wang et al., 2015; Fürst-Jansen et al., 2020). Phytohormones are well-established regulators that are involved in nearly every aspect of land plant life including plant development and the response of plants to changes in their abiotic and biotic environment. Phytohormone biology can be divided into three parts (Eklund et al., 2018; Sun et al., 2019; Blazquez et al., 2020; Yang et al., 2020): metabolism (biosynthesis and degradation), transport, and perception and signaling. While some components, such as polyamine and signaling peptides, are sometimes considered plant growth regulators, there are only nine different groups of chemicals that are traditionally referred to as plant hormones (Guillory and Bonhomme, 2021). These have been characterized into three big groups according to the nature of their signaling pathways: (1) F-Box protein-mediated signaling (auxins (Auxs), jasmonic acid (JA), salicylic acid (SA), gibberellins (GAs), strigolactones (SLs)), (2) Two-component signaling pathway (TCS)-related (cytokinins (CKs) and ethylene) and (3) others, those with unrelated signaling pathways [abscisic acid (ABA), brassinosteroids (BRs)] (Bowman et al., 2019). The first comprehensive studies looking into the evolution of phytohormone signaling were published more than a decade ago, focusing on cytokinin and auxin, respectively (Pils and Heyl, 2009; de Smet et al., 2011). The availability of an increasing number of sequenced genomes has allowed this type of study to become more comprehensive, covering several phytohormones at one time (Wang et al., 2015; Bowman et al., 2019; Blazquez et al., 2020). Recent reviews have covered the evolution of the F-box protein hormone pathways (Blazquez et al., 2020), ABA (Eklund et al., 2018; Sun et al., 2019: Yang et al., 2020), and ethylene (Ju et al., 2015; Bidon et al., 2020; Binder, 2020). Thus, this review will focus on the evolution of different aspects of cytokinin biology.

## Cytokinin metabolism and its evolution

Cytokinins play a crucial role not only in plant development but also in the reaction of plants to abiotic and biotic stress (Kieber and Schaller, 2018; Cortleven et al., 2019). First discovered in the 1950s by Miller and colleagues in Skoog’s lab (Miller et al., 1955), cytokinins are adenine derivatives, which can be classified into two groups, depending on whether the *N^6^-*sidechain is aromatic or an isopentenyl (Heyl et al., 2006; Frébort et al., 2011; Lindner et al., 2014). Cytokinins are found in cyanobacteria, plants, algae, and many other organisms. While one could speculate that they are just degradation products of nucleic acids – as in the case of their original discovery (Miller et al., 1955) - analysis of the cytokinin content over time showed that at least in cyanobacteria, amoeba, and *Viriplantae*, the types and the concentrations of the different cytokinins are highly regulated (Gajdosova et al., 2011; Žižková et al., 2017; Aoki et al., 2019). In fact, several experiments with a large variety of different algae (e.g. *Acutodesmus obliquus, Laurencia catarinensis)*, have shown that, although no canonical cytokinin receptor genes are present in the genomes, they respond to treatment with phytohormones, especially auxin, and cytokinin, with increased cell growth and/or metabolic activity (Lu and Xu, 2015; Renuka et al., 2017; Piotrowska-Niczyporuk et al., 2018; Piotrowska-Niczyporuk et al., 2020; Araújo et al., 2021). This could point to the existence of an alternative, not-yet-described signaling pathway, or a direct interaction between cytokinins and effector proteins. In the later case, cytokinins would function as allosteric regulators.

The rate-limiting step of cytokinin synthesis is catalyzed by isopentenyl transferases (IPTs), which can use either tRNA (tRNA pathway) or ATP/ADP/AMP (adenylate pathway) as a substrate (Lindner et al., 2014; Frébortová and Frébort, 2021). The tRNA pathway mainly leads to the production of *cis-*Zeatin (cZ) and is believed to be the evolutionarily older pathway of the two (Frébort et al., 2011; Frébortová et al., 2015). Looking at the phylogenetic history of IPT genes, tRNA-type IPTs, which are present in bacteria, algae, and land plants, are evolutionarily older than the adenylate-type, which are only found in land plants. However, the evolution of this group of key enzymes is complex and characterized by a potential horizontal gene transfer (HGT) and multiple, independent emergences (Frébort et al., 2011; Lindner et al., 2014; Frébortová et al., 2015; Nishii et al., 2018). Interestingly, the adenylate pathway was long thought to be specific to Angiosperms but there is evidence of an alternative cytokinin production pathway - independent of tRNA – in the moss *Physcomitrium patens* (Lindner et al., 2014). Recently it was proposed that tRNA IPTs are mainly used to produce *cis*-Zeatin (cZ), which mainly acts in housekeeping, i.e., the maintenance of basic cell function while adenylate IPT, which mainly produces tZ (*trans*-Zeatin) and IP (Isopentenyl)-type cytokinin, play regulatory roles (Wang et al., 2020b). This hypothesis was supported by the difference in expression patterns: while the expression of tRNA-type IPTs is more or less constitutive in Angiosperms, adenylate IPTs are highly regulated in their expression (Wang et al., 2020b).

Cytokinins can be inactivated reversibly by *N-* or *O-*glycosylation (Heyl et al., 2006). However, it has been shown for *N*-glucosides that they can trigger at least a partial cytokinin response in some species (Pokorná et al., 2020). In contrast, the cleaving of the aromatic or isopentenyl side chain from the adenine backbone is permanent (Werner et al., 2006). This cleavage is catalyzed by a group of enzymes called cytokinin dehydrogenase (CKXs). The expression of most *CKXs* in the model plant Arabidopsis is regulated, and their overexpression can lead to severe phenotypes (Werner et al., 2001; Schmülling et al., 2003). No CKX activity was detected in cyanobacteria or algae, although sequences for *CKX* genes are present in some of the cyanobacteria genomes investigated (Frébortová et al., 2015; Žižková et al., 2017; Dabravolski and Isayenkov, 2021). However, all land plants investigated so far encode for *CKX* genes and many have been shown to be functional (Frébort et al., 2011; Wang et al., 2020a). How and when CKXs emerged in land plants is not clear, as they are found in only a few algae (e.g. *Chara braunii*). It has been speculated that they might have been gained *via* horizontal gene transfer from bacteria (Schmülling et al., 2003; Dabravolski and Isayenkov, 2021). It has been proposed that *CKXs* can be divided into two groups, which is analogous to the model proposed for the *IPT* genes. One group degrades cZ and thus is involved in the housekeeping functions of cytokinin. The expression of these CKXs does not appear to be highly regulated (Wang et al., 2020a). The second, more recently evolved group breaks down IP and tZ-type cytokinins and therefore plays a more regulatory role. Thus, their expression is under a tightly regulated regime (Wang et al., 2020a). A similar idea of a two-tiered system, involving not only different members of cytokinin metabolism but also components of the signaling pathway, has been proposed more recently (Hluska et al., 2021). These theories for the evolutionary history and function of different types of IPT and CKX proteins are indeed intriguing. However, more experimental evidence in different plant species is needed to corroborate these ideas.

## Evolution of cytokinin signaling

The cytokinin signal is perceived and transmitted by a variant of the Two-Component System (TCS). The classical TCS consists of two components - hence the name - a receptor kinase, which autophosphorylates upon the perception of the signal, and a response regulator, which after activation through phosphorylation by the receptor mediates the downstream response, e.g., *via* regulation of transcription (West et al., 2001). In bacteria, a variant of this system is used in which the phosphorylation signal is first transferred within the receptor from the histidine kinase to a receiver domain and then transmitted to a histidine phosphotransfer protein (HPt). HPts in turn activate their cognate response regulators (West et al., 2001). The TCS mediating the cytokinin signaling system is best characterized in the model plant Arabidopsis (Hwang and Sheen, 2001; Gruhn and Heyl, 2013; Kieber and Schaller, 2018). Cytokinins are perceived by Cyclase/Histidine Kinase Associated Sensory Extracellular (CHASE) domain-containing hybrid histidine kinase receptors (CHKs) either at the plasma membrane and/or at the ER membrane (Wulfetange et al., 2011; Romanov et al., 2018; Antoniadi et al., 2020; Kubiasová et al., 2020). After intramolecular phosphorylation, the signal is transferred to HPt proteins, which shuttle between the cytokinin and the nucleus where they phosphorylate response regulators (Hutchison et al., 2006; Punwani et al., 2010). There are two different kinds of response regulators involved in cytokinin signaling in plants: i. the type-B response regulators (RRB), which are Myb transcription factors and activate the transcription of a group of target genes, one of which is the ii. type-A response regulators (RRA). The RRAs negatively regulate the signaling pathway, most likely by competing with the RRB for the phosphorylation signal from the HPts (Hwang and Sheen, 2001; Gruhn and Heyl, 2013).

Currently, there are more than 300,000 different TCS identified, and they are found across all kingdoms, with the notable exception of animals (Papon and Stock, 2019). While the vast majority of those systems serve signaling within the given species, they have also been found to serve interspecies interaction, in both pathogenic and symbiotic interactions (Cortleven et al., 2019; Papon and Stock, 2019; Bidon et al., 2020). Originating in prokaryotes, the TCS is thought to have been transferred to eukaryotes either *via* endosymbiosis or horizontal gene transfer (Schaller et al., 2011; Spíchal, 2012; Rashotte, 2021).

## Cytokinin receptors

Cytokinin perception, whether on the plasma or the ER membrane, is mediated (perceived) by the CHASE domain, and all known cytokinin receptors possess such a protein domain. However, CHASE domains are also found in a wide variety of other receptors (Schaller et al., 2011, Kabbara et al., 2018). Therefore, the mere presence of the CHASE domain alone does not automatically qualify a protein to be a cytokinin receptor. Proteins containing all the canonical domains of CHKs can be found encoded in the genomes of a wide variety of different streptophyte algae (Bidon et al., 2020). In fact, CHASE domain-containing hybrid histidine kinase receptors are found in bacteria, amoeba, and fungi, but experimental evidence that those proteins are functional in cytokinin perception is missing in most cases (Bidon et al., 2020). This is especially important as even identical protein folds do not mean that the respective proteins bind the same ligand (Papon and Stock, 2019). A good example of this phenomenon is the members of the subfamily of CHKs, which have been discovered in the moss *Physcomitrium patens*. This model organism for the bryophyte branch of land plants is unusual, as it encodes 11 CHKs in its genome. Three of those CHKs form a clade with the well-characterized cytokinin receptors from Tracheophytes, while the other eight form a separate clade (Gruhn et al., 2014). At least one of those eight unusual CHKs was shown to possess some property of cytokinin receptors but did not display the same level of specificity for different cytokinins as compared to the well-characterized cytokinin receptor AHK4 from *A. thaliana.* Subsequent analysis, both biochemically and *in planta*, showed that members of this subfamily most likely do not play a role in the canonical cytokinin signaling in *P. patens* (Gruhn et al., 2014; von Schwartzenberg et al., 2016; Lomin et al., 2021). Interestingly, CHKs from streptophyte algae and many bryophytes do not form a clade with the experimentally proven cytokinin receptor, but with this unusual subfamily of CHKs from *Physcomitrium* (Gruhn et al., 2014; Bowman et al., 2017; Aki et al., 2019). Thus, it might be considered that CHKs of the algae and bryophytes, forming a clade with the subfamily of CHKs from *Physcomitrium*, might not function as *bona fide* cytokinin receptors, but have other functions. Only during the evolution of vascular plants, the main task of CHKs became the perception of cytokinin. A recent study looking at the cytokinin binding properties of CHKs from *Physcomitrium*, *Selaginella*, and *Pinus* found that there is an increase in specificity for tZ over cZ during plant evolution (Lomin et al., 2021). The authors speculate that in the last common ancestor of tracheophytes and bryophytes, there were few tasks for cytokinin and thus, the cytokinin production *via* tRNA was sufficient. During the course of evolution, as more tasks have become cytokinin-dependent, iP and *t*Z became produced *via* the adenylate pathway and receptors evolved to be more specific for *t*Z perception (Lomin et al., 2021).

While the numbers of the members of other protein families of the cytokinin signaling pathway increase steadily during the diversification of vascular plants - probably due to numerous rounds of whole genome duplication (WGD) - the number of CHKs remains near-constant (**Table 1**). This is most likely due to selective gene loss in the CHKs (Kaltenegger et al., 2018). The exact phylogenetic relationship of CHKs of non-seed plants and gymnosperms is hard to resolve, but within the angiosperm, three clades are clearly distinguishable – referred to by their members from Arabidopsis as AHK2-, AHK3- and AHK4-clades, respectively (Pils and Heyl, 2009; Kaltenegger et al., 2018).

**Table 1:** Number of key cytokinin metabolic and signaling genes in different plant species.

Species	IPT - tRNA	IPT - Adenyl	CKX	CHK	HPT (AHPT/ PHPT)	RRB	RRA	Reference
*Arabidopsis thaliana*	2	7	7	3	6 (5/1)	11	10	Nishii et al., 2018 Heyl et al., 2006
*Oryza sativa*	2	7	11	5	5 (2/3)	13	10	Ghosh et al., 2018 Tsai et al., 2012
*Amborella trichopoda*	2	2	5	2	4 (3/1)	6	4	Nishii et al., 2018 Wang et al., 2020c Kaltenegger et al., 2018
*Picea abies*	4	1	10	2	7(5/2)	7	12	Wang et al., 2020a Wang et al., 2020b Kaltenegger et al., 2018
*Sellaginela moellendorfii*	1	0	2	2	2 (2/0)	10	3	Nishii et al., 2018 Wang et al., 2020b Kaltenegger et al., 2018
*Physcomitrum patens*	8	0	5	3 (+8)	2 (2/0)	5	7	von Schwartzenberg et al., 2016 Nishii et al., 2018 Wang et al., 2020b Gruhn et al., 2014
*Marchantia polymorpha*	2	0	2	2	1 (1/0)	1	1	Nishii et al., 2018 Wang et al., 2020b Bowman et al., 2017 Aki et al., 2019
*Anthoceros agrestis*	3	3	2	1	1 (1/0)	1	1	Li et al., 2020
*Zygema cylindrium*	NA	NA	NA	5	1 (1/0)	2	0	Feng et al., 2023
*Spirogyra pratensis*	NA	NA	NA	4	NA	1	1	de Vries et al., 2020
*Chara braunii*	1	0	1	2	1 (1/0)	0	1	Wang et al., 2020c Nishiyama et al., 2019
*Klebsormidium nitens*	1	0	0	9	1 (1/0)	1	3	Žižková et al., 2017 Nishii et al., 2018 Kaltenegger et al., 2018
*Chlorokybus atmophyticus*	3	0	0	1	1 (1/0)	1	0	Wang et al., 2020c
*Mesostigma viride*	2	0	0	2	1 (1/0)	1	1	Wang et al., 2020c
*Chlamydomonas rheinhardtii*	1	0	0	0	1 (1/0)	1	0	Nishii et al., 2018; Wang et al., 2020b Pils and Heyl, 2009

IPT (tRNA) Isopentenyltransferase (tRNA-type); IPT (adenyl) Isopentenyltransferase (adenylate-type); CHK CHASE-domain containing histidine kinase; HPT histidine phosphotransfer protein; AHPT authentic histidine phosphotransfer protein; PHTP pseudo histidine phosphotransfer protein; RRB response regulator type-B; RRA response regulator type-A; NA data not available.

## Histidine phosphotransfer proteins

In contrast to CHKs, the next type of protein in the signaling chain, the HPts, can be found across many kingdoms (Singh et al., 2021). Most algae have one or two HPts encoded in their genomes, and all of these contain the canonical His residue and thus should be functional (Gruhn et al., 2014). This is also true for bryophytes (**Table 1**). In contrast to the evolutionary fate of the CHKs, some of the extra copies of the HPts after whole genome duplications were not lost and so a small but steady increase in the number of HPts can be detected throughout plant evolution (Gruhn et al., 2014; Kaltenegger et al., 2018). Interestingly, starting with the gymnosperms, a variant of the HPts appears. In these variants, the canonical His residue is replaced by a different amino acid, thus making the protein unable to accept or transfer the phosphoryl residue. In Arabidopsis, the pseudo HPt (AHP6) functions as a negative regulator in the cytokinin signaling pathway (Mähonen et al., 2006). However, a recent study of canonical and pseudo HPts from rice showed that pseudo HPts can also play a positive role in cytokinin signaling and thus function differently than those from Arabidopsis (Vaughan-Hirsch et al., 2021). More such studies of HPts from different species are needed to fully understand the role that pseudo HPts play in cytokinin signaling.

## Response regulators

For the two types of response regulators (RRA and RRB) the evolutionary pattern is more similar to the HPts than the CHKs. But there are also differences between the two types that are quite striking. RRBs are present in most streptophyte algae, and in land plants, but also in chlorophyte algae (**Table 1**). In general, the number of RRB per species is relatively low. This starts to change during the evolution of land plants and might be related to the increasing number of Whole Genome Duplications (WGDs), which in the case of the RRB were not followed by gene loss (Kaltenegger et al., 2018). It seems to be that there were at least four RRBs present in the last common ancestor of monocots and dicots, but subsequent WGDs and gene retentions increased those numbers to 10-20, depending on the species (Kaltenegger et al., 2018). In the case of the RRAs, there are none found in Chlorophyceae algae, and also some of the more closely related charophytes do not encode these negative regulators of cytokinin signaling in their genomes. However, in all land plants RRAs are present (**Table 1**). Similar to the RRBs, the major fate of RRAs after WGD seems to be gene retention, leading to a steady increase in the number of RRAs during land plant evolution (Kaltenegger et al., 2018).

## Cytokinin signaling beyond the TCS

However, the TCS seems to be not the only way that the cytokinin signal can be perceived and transduced. Some species of rice encode CHASE-domain receptor serine/threonine kinases (CHARK). As the name indicates these proteins combine a CHASE domain with a serine/threonine kinase domain (Du et al., 2007). Although it does not contain a histidine kinase domain, it can complement Arabidopsis cytokinin receptor mutants (Han et al., 2004; Halawa et al., 2021). The fact that primary cytokinin response genes have the same expression level in wild-type plants as in CHARK-complemented receptor mutants could hint at a direct interaction of CHARK with the canonical TCS (Halawa et al., 2021). However, the signaling chain emanating from CHARK needs to be established experimentally. One alternative could be the phospholipase D-mediated pathway. In the past, a close and rapid connection between cytokinin treatment and a phospholipase D signaling pathway has been proposed (Romanov et al., 2002; Kravets et al., 2009). In addition, a proteomic approach to identify proteins of the early cytokinin response showed many Ser/Thr kinase-regulated proteins, which are known to play a role in phospholipase D signaling (Cerny et al., 2011). So, while most of the cytokinin research points to the TCS as the major player in cytokinin signaling, other types of signaling systems might play a role as well, especially in less well-investigated species.

## Model for the evolution of the cytokinin signaling system

**Figure 1:**
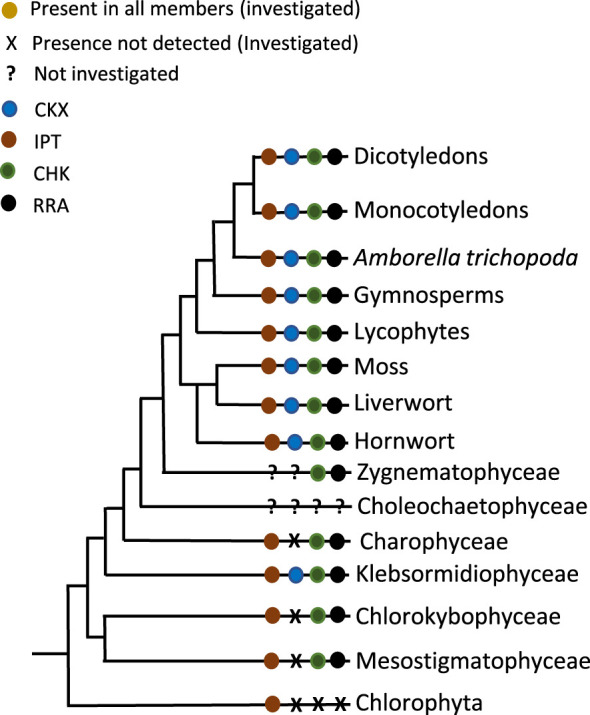
Presence or absence of the components of cytokinin biology across the *Viridiplantae* (for *Choleochaetophyceae* no genome data were available).

In summarizing the current state of knowledge about cytokinin biology, a model for the origin and evolution of the cytokinin signaling system can be proposed (**Figure 1**). Cytokinins are degradation products of nucleic acids and therefore can be found in all organisms. However, the cytokinin content in many organisms changes in a controlled manner, and even species without CHKs or RRAs react to cytokinin on a metabolic and cellular level (Žižková et al., 2017; Piotrowska-Niczyporuk et al., 2020). This indicates there must be another way by which cytokinins control cellular processes. The activity of many enzymes regulating metabolic pathways is controlled by small molecules. Thus, it is conceivable that cytokinin can exert direct control of metabolic processes *via* regulating key enzymes, even in the absence of a dedicated signaling pathway – in effect functioning as an allosteric regulator. Experiments with brassinolide treatment of *Asterionella formosa*, a diatom, hinted at such a direct effect on glyceraldehyde-3-phosphate dehydrogenase, a key enzyme in glycolysis and also in the Benson - Calvin cycle (Mekhalfi et al., 2012). This means of regulation might be sufficient in unicellular organisms, where there are relatively few regulated activities. Once the complexity of the organism increases, both morphologically and biochemically, more complex regulation becomes necessary. This could have occurred in several steps for both, the hormone metabolism and the hormone signaling, respectively (**Table 1**). On the metabolic side, during the evolution of land plants, there is a shift from the tRNA-based production of mainly cZ to the adenylate-type pathway for the production of mainly tZ and iP- type cytokinins. The adenylate IPTs seemed to have evolved several times independently of each other from tRNA-type predecessors, which might be an indication of the necessity of such a mode of cytokinin production (Nishii et al., 2018; Wang et al., 2020b). This process is accompanied by the appearance of an effective and dedicated degradation pathway in the form of CKX enzymes. During the evolution of the land plants, numerous rounds of WGD led to an increase in the copy number of these key enzymes for cytokinin metabolism (**Table 1**).

On the signaling side, the presence of the hormone could have been first “perceived” by key enzymes that were bound and allosterically regulated by the hormones. During the evolution of the streptophytes a pre-existing gene regulatory network, consisting of HPts and RRB, could have been repurposed by adding a different receptor, in this case, the CHKs, to it. This type of process has been proposed for other pathways before (Pires et al., 2013; Fürst-Jansen et al., 2020). In the beginning, such a pathway might not have been used for cytokinin sensing, but for the detection of other inputs, such as pH (Lomin et al., 2021). This seems likely as cytokinin sensing has so far been experimentally shown only for those CHKs that form a clade with *bona fide* cytokinin receptors of Arabidopsis and rice. The CHKs of streptophyte algae and bryophytes form a different clade from the CHK of the well-characterized cytokinin receptors. (Gruhn et al., 2014; Wang et al., 2015; Kaltenegger et al., 2018; Li et al., 2023). During the early phase of land plant evolution, cytokinin-binding CHKs and RRAs evolved – as they are found in all extant land plants. Thus, the first complete cytokinin signaling pathway – as we know it from the model land plants, such as Arabidopsis and rice – evolved. The next steps in the evolution of cytokinin signaling involved specialization and subfunctionalization of the components of this signaling system (Kaltenegger et al., 2018; Lomin et al., 2021).

## Conclusion: general principles in origin and evolution of cytokinin biology

1.) Cytokinins are present before the establishment of their cognate signaling pathway.

Cytokinins, like other phytohormones are found in many green algae and plants - regardless of the presence of the dedicated signaling pathway known from angiosperm model plants, e.g., auxin (Mutte et al., 2018), cytokinin (Gruhn et al., 2014), ethylene (Ju et al., 2015). In fact, completely different signaling pathways for cytokinin might exist, or cytokinins might work as allosteric regulators of key enzymes in specific metabolic pathways. A proteomic approach identified numerous cytokinin-binding proteins that could be involved in such pathways (Simerský et al., 2017).

2.) Downstream signaling pathways are present before the appearance of receptors.

It has long been proposed that new regulatory pathways are made by using existing gene regulatory pathways and connecting them to a novel input (plugin) (Davidson and Erwin, 2006; Pires et al., 2013). Thus, it is not surprising that in cytokinin (and other hormones) signaling, the downstream part of a pathway is present before the appearance of a functional signal perception module and might fulfill functions different from hormone signaling (**Table 1**) (Pils and Heyl, 2009; Wang et al., 2015; Bowman et al., 2017; Briones-Moreno et al., 2017; Mutte et al., 2018; Sun et al., 2019; Blazquez et al., 2020; Fürst-Jansen et al., 2020; Sun et al., 2020).

3.) Receptors are often the last step to completing a pathway.

Connected to the previous points, it is often the case that receptors are present, but are not yet able to bind the cytokinin or to convert the binding of the ligand into the activation of the cognate signaling pathway. This has been shown for other hormones, the most prominent example being the GA receptors. They are present in *P. patens* and interact with the downstream components of the GA signaling pathway but do so without the binding of GA (Yasumura et al., 2007). A similar situation was observed more recently for ABA (Fürst-Jansen et al., 2020) and jasmonic acid (Hu et al., 2023).

4.) The specificity of the receptor-cytokinin interaction increases in more recently evolved lineages.

Once the complete cytokinin signaling pathway was in place, the receptors displayed a low specificity for the different cytokinins. During evolution, the specificity of the receptor-ligand interaction increased for the different types of the cytokinin found in plants (Lomin et al., 2021). Similarly, a shift in binding preferences for different JA metabolites has been observed in JA signaling (Monte et al., 2019; Hu et al., 2023). This might be true for other plant hormones as well.

5.) Different parts of a signaling pathway can be under different evolutionary constraints.

Another fascinating aspect of the evolution of cytokinin and other phytohormones is that the different parts of the signaling system are under different evolutionary constraints. It is often found that the number of receptors is relatively constant while downstream components increase in number (**Table 1**) (Kaltenegger et al., 2018; Blazquez et al., 2020).

6.) Horizontal gene transfer and whole genome duplications could be major drivers for evolutionary novelties in cytokinin biology.

What are the major mechanisms for producing the evolutionary novelties found during the evolution of cytokinin signaling? One major factor could be the transfer of genetic material from bacteria/viruses *via* horizontal gene transfer (HGT). In fact, it has been speculated that the TCS system was delivered to a photosynthetic organism during the primary endosymbiosis of cyanobacteria (Pils and Heyl, 2009; Rashotte, 2021). Horizontal gene transfer has also been suggested as a source for the CHKs of brown algae, as the closest relative of those CHASE domains is found in mega viruses (Pils and Heyl, 2009; Kabbara et al., 2019). Also, for the major cytokinin degrading enzyme, CKX, a HGT from bacteria was proposed, as this type of protein is found in bacteria and all land plants, but only sporadically in the genomes of algae (Schmülling et al., 2003; Dabravolski and Isayenkov, 2021, **Table 1**). Gene duplication, often as a consequence of WGD, is also considered to be a major source of evolutionary novelties in phytohormone biology, such as the pseudo HPts of Tracheophytes (Mähonen et al., 2006; Gruhn et al., 2014; Vaughan-Hirsch et al., 2021). But such duplication can also be used in the pathway of a different hormone, e.g., it has been proposed that the JA receptor (*Coronatine Insensitive, COI*) originates from a gene duplication of the auxin receptor (*Transport Inhibitor Response, TIR*) (Bowman et al., 2017; Mutte et al., 2018). The role of WGD in the formation of the cytokinin signaling pathway found in plants as discussed above is another example of the importance of this phenomenon in plant evolution.

Each of these key points, by itself or in combination with each other, is important for understanding the origin and evolution of cytokinin signaling.

## Author contributions

Both authors wrote the manuscript. All authors contributed to the article and approved the submitted version.
