# Supplementary material for: The origin and early evolution of cytokinin signaling
Source: Front Plant Sci. 2023 Jun 28;14:1142748. doi: 10.3389/fpls.2023.1142748 (PMC10338860; doi:10.3389/fpls.2023.1142748)
Supplement: Supplementary file 1 [file DataSheet_1.pdf]

Supplemental Table1 S1: Species represented in Figure 1

Plant Group	Species	Reference
Dicotyledons	*Arabidopsis thaliana*<br>*Brassica rapa*<br>*Malus domestica*<br>*Solanum lycopersicum*<br>*Solanum tuberosum*	Phytozome (https://phytozome-next.jgi.doe.gov/)
Monocotyledons	*Oryza sativa*<br>*Zea mays*<br>*Sorghum bicolor*	Phytozome (https://phytozome-next.jgi.doe.gov/)
Amborella trichopoda	*Amborella trichopoda*	Nishii *et al.,* 2018; Kaltenegger *et al.,* 2018
Gymnosperms	*Pinus trichocarpa*<br>*Picea abies*	Pils and Heyl, 2009; Wang *et al.,* 2020 a, b; Kaltenegger *et al.*, 2018
Lycophyta	*Selaginella moellendorfii*	Nishii *et al.,* 2018; Kaltenegger *et al.*, 2018; Wang *et al.,* 2020 b
Moss	*Physcomitrium patnes*	Rensing *et al.*, 2008; Nishii *et al.,* 2018
Hornwort	*Anthoceros agrestis*	Li *et al.,* 2023
Liverwort	*Marchantia polymorpha*	Nishii *et al.,* 2018: Bow
Zygnematophyceae	*Zygema cylindrium*<br>*Spirogyra pratensis*	Feng *et al.,* 2023
Coleochaetophyceae		No data available
Charophyceae	*Chara braunii*	Wang *et al.* 2020c<br>Nishiyama *et al.* 2018
Klebsormidiophyceae	*Klebsormodium nitans*	Zizkova *et al.* 2017<br>Nishii *et al.* 2018<br>Kaltenegger *et al.* 2018
Chlorokybophyceae	*Chlorokybus atmophyticus*	Wang *et al.* 2020c
Mesostimatophyceae	*Mesostigma viride*	Wang *et al.* 2020c
Chlorophyta	*Chlamydomonas rheinhardtii*<br>*Osteococcus taurii*<br>*Volvox craterii*	Phytozome (https://phytozome-next.jgi.doe.gov/)<br>Pils and Heyl, 2009, Wang et al.c, 2020
